# Supplementary material for: Co-activation of Akt, Nrf2, and NF-κB signals under UPRER in torpid Myotis ricketti bats for survival
Source: Commun Biol. 2020 Nov 11;3:658. doi: 10.1038/s42003-020-01378-2 (PMC7658203; doi:10.1038/s42003-020-01378-2)
Supplement: Supplementary file 1 — Supplementary Information [file 42003_2020_1378_MOESM1_ESM.docx]

**Supplementary Figures**


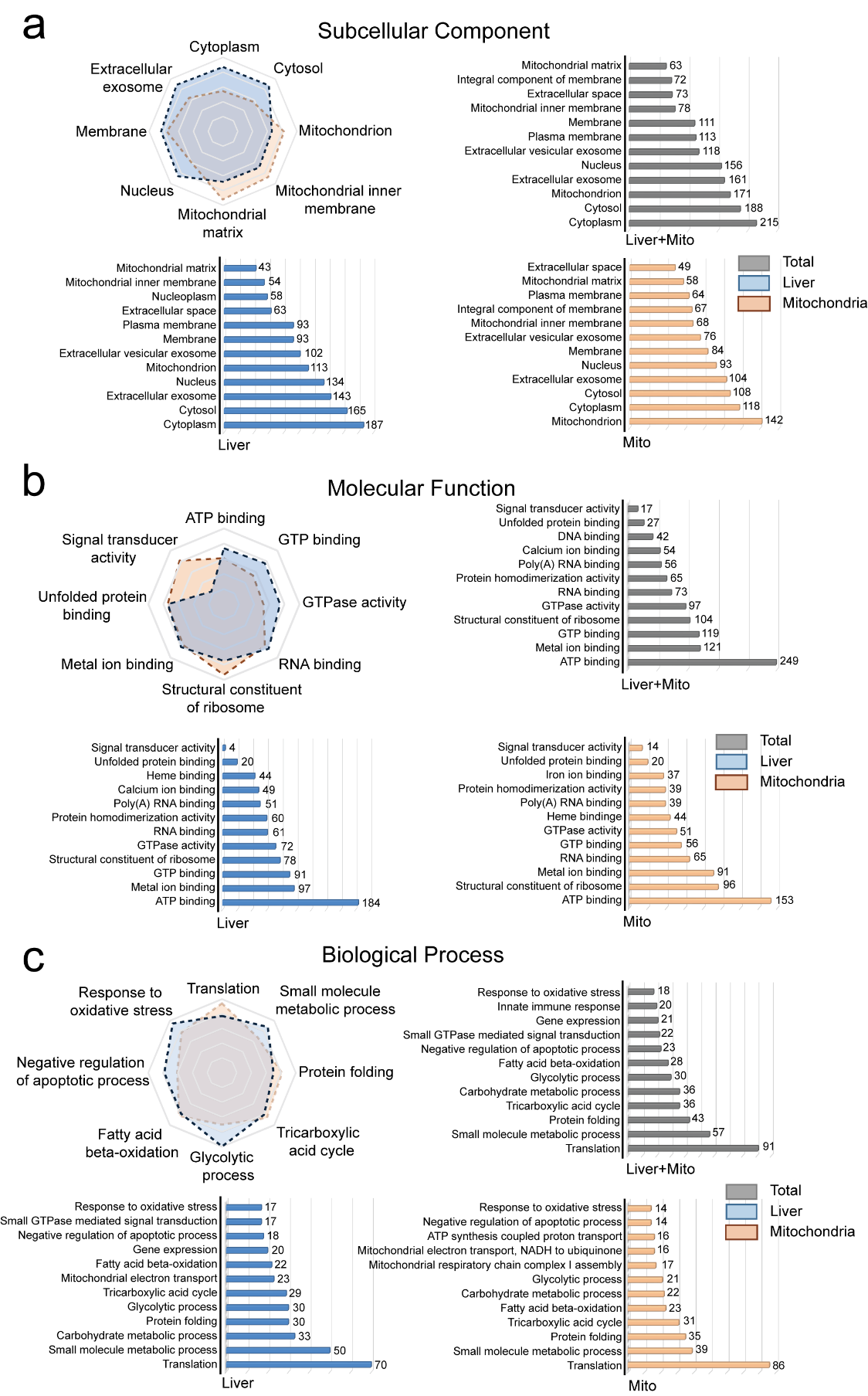


**Supplementary Fig. 1. Results of GO analysis**. **a-c** Enrichment analysis by Gene Ontology (GO) according to Subcellular component (**a**), Molecular function (**b**), Biological process (**c**).


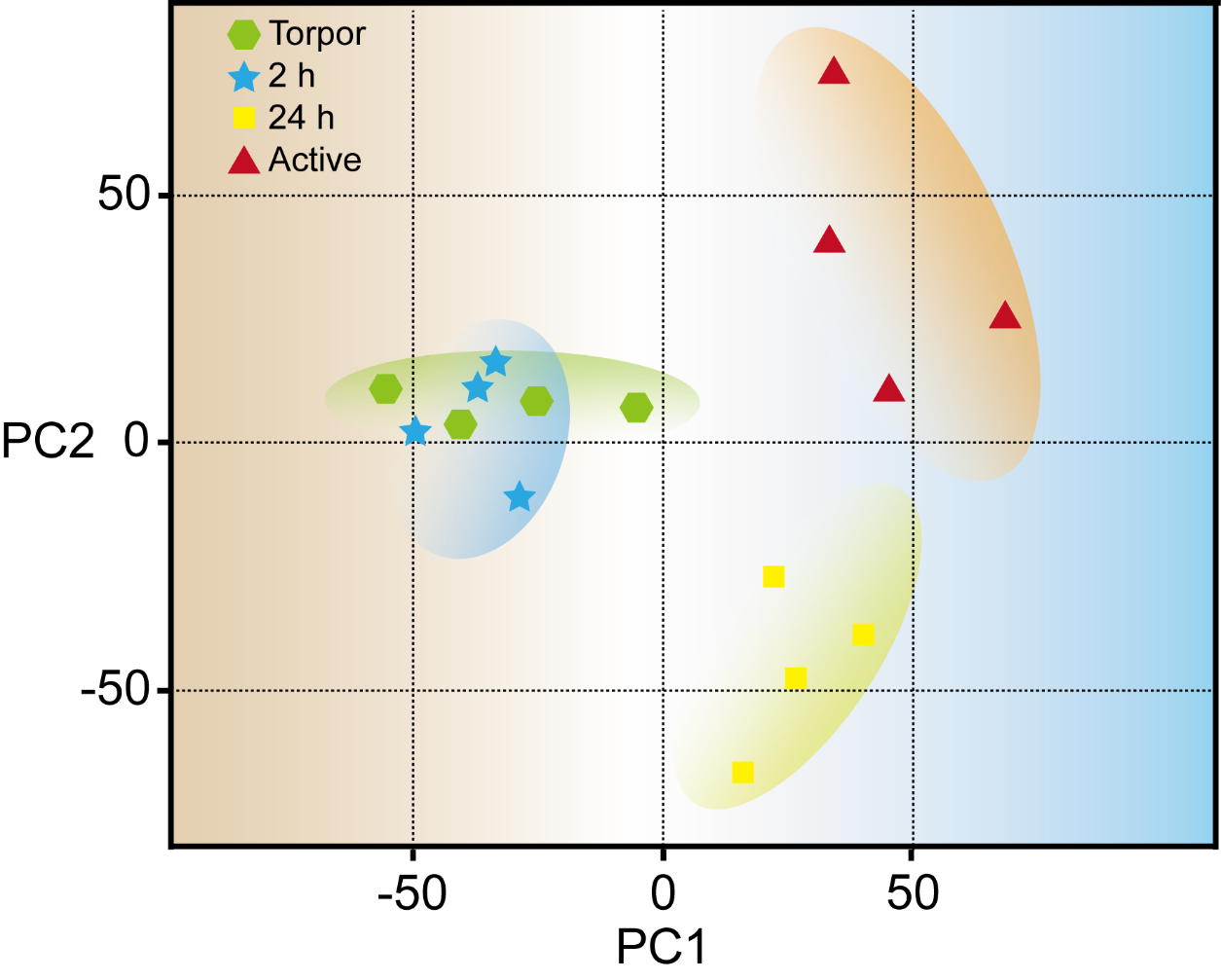


**Supplementary Fig. 2.** **Principal Component Analysis of 4 states of bats.** PC1 and PC2 are two top-ranked components corresponding 54% of variation in dataset. 12 bat samples are shown, including 4 each from torpor (blue), 2 h-active (green), 24 h-active (orange) and Active (red) groups.


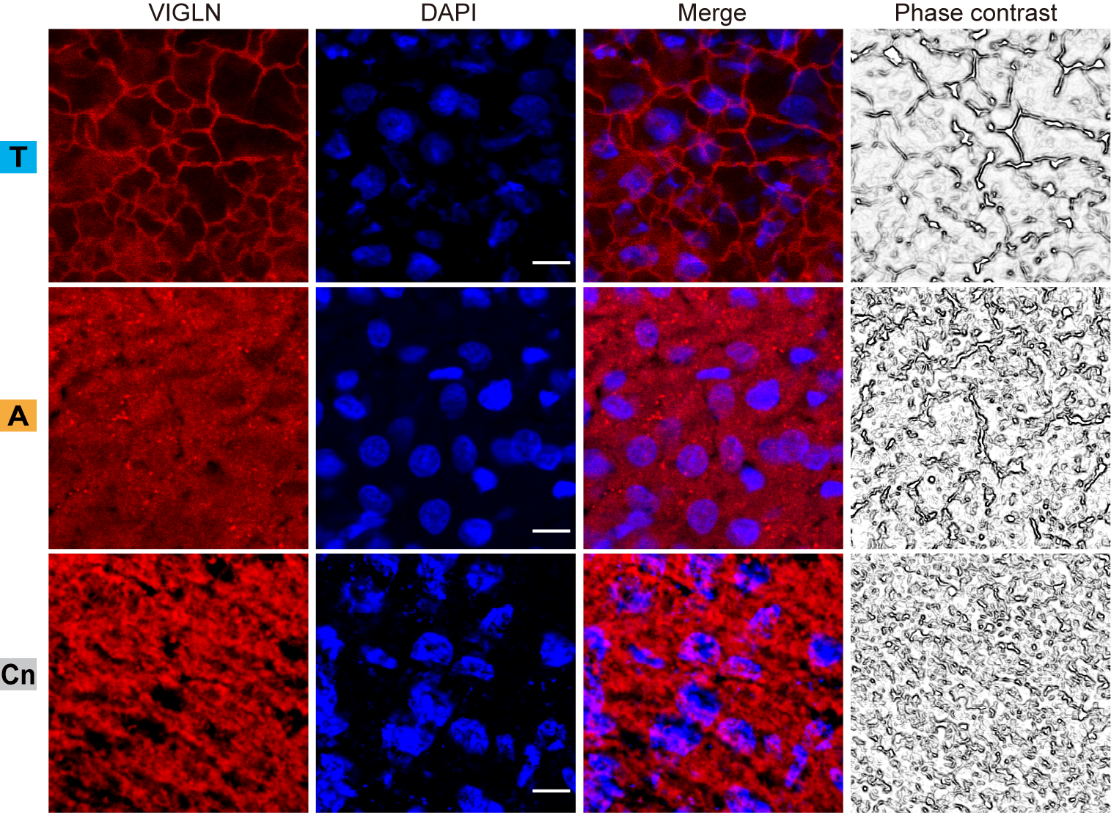


**Supplementary Fig. 3.** Images of immunofluorescence microscopy of VIGLN (in red) and nuclei (in blue) and phase contrast microscopy (Scale bars: 20 μm). T, A, and Cn represent samples of torpid bats, active bats, and control mice, respectively.


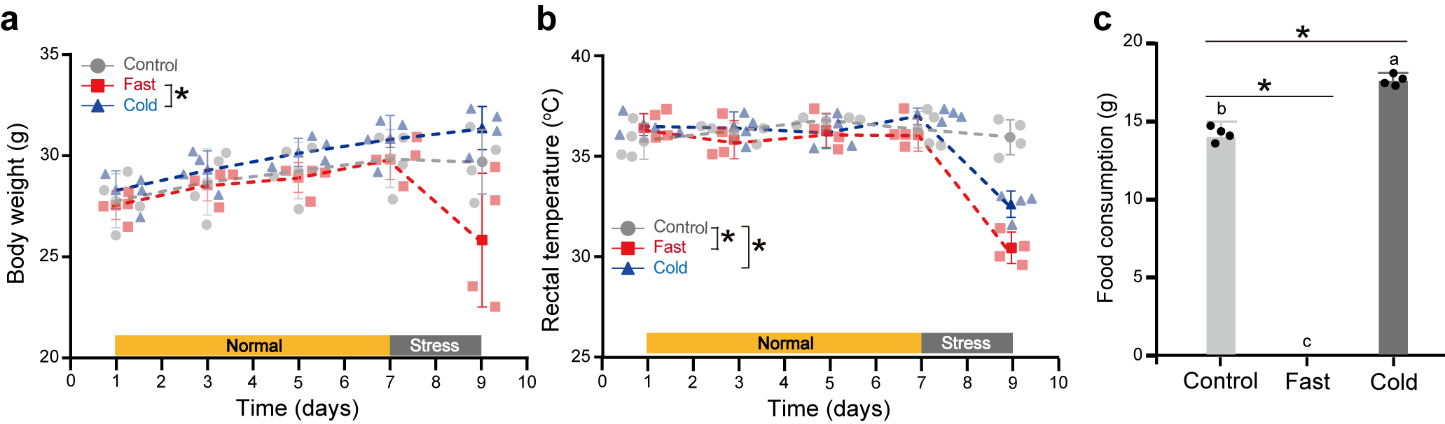


**Supplementary Fig. 4.** **Life index of cold stimulated and fasted mice**. **a-c** Body weight (**a**), body temperature (**b**) and food consumption (**c**). Data are expressed as means ± SD (n = 4), ^*^*P* < 0.05.


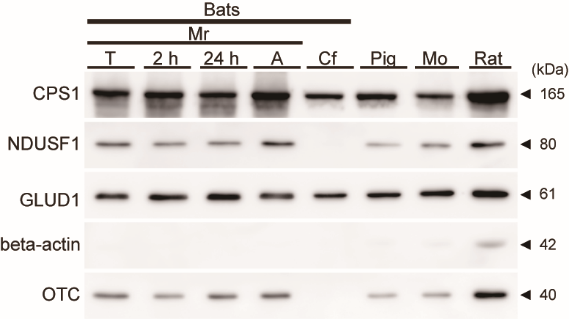


**Supplementary Fig. 5.** Western blotting of mitochondrial proteins CPS1, NDUSF1, GLUD1, OTC and the cytoplasmic protein beta-actin. Mitochondrial proteins were prepared from *Cynopterus sphinx* bats, pig, moue, and rat and *Myotis ricketti* bats at torpor (T), 2 hours arousal (2 h), 24 hours arousal (24 h), and active (A) sates.


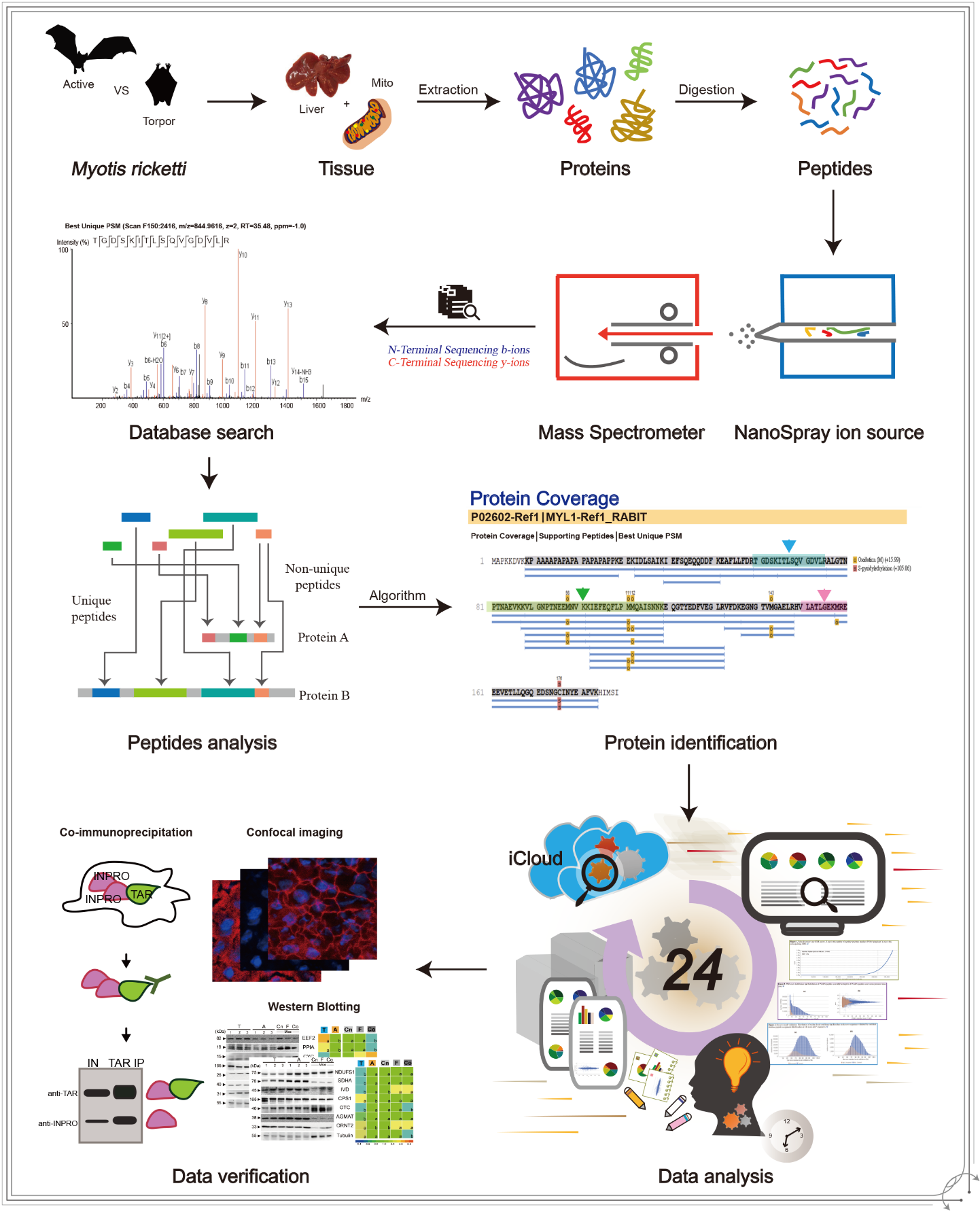


**Supplementary Fig. 6.** Flow chart of Shotgun Proteomics.


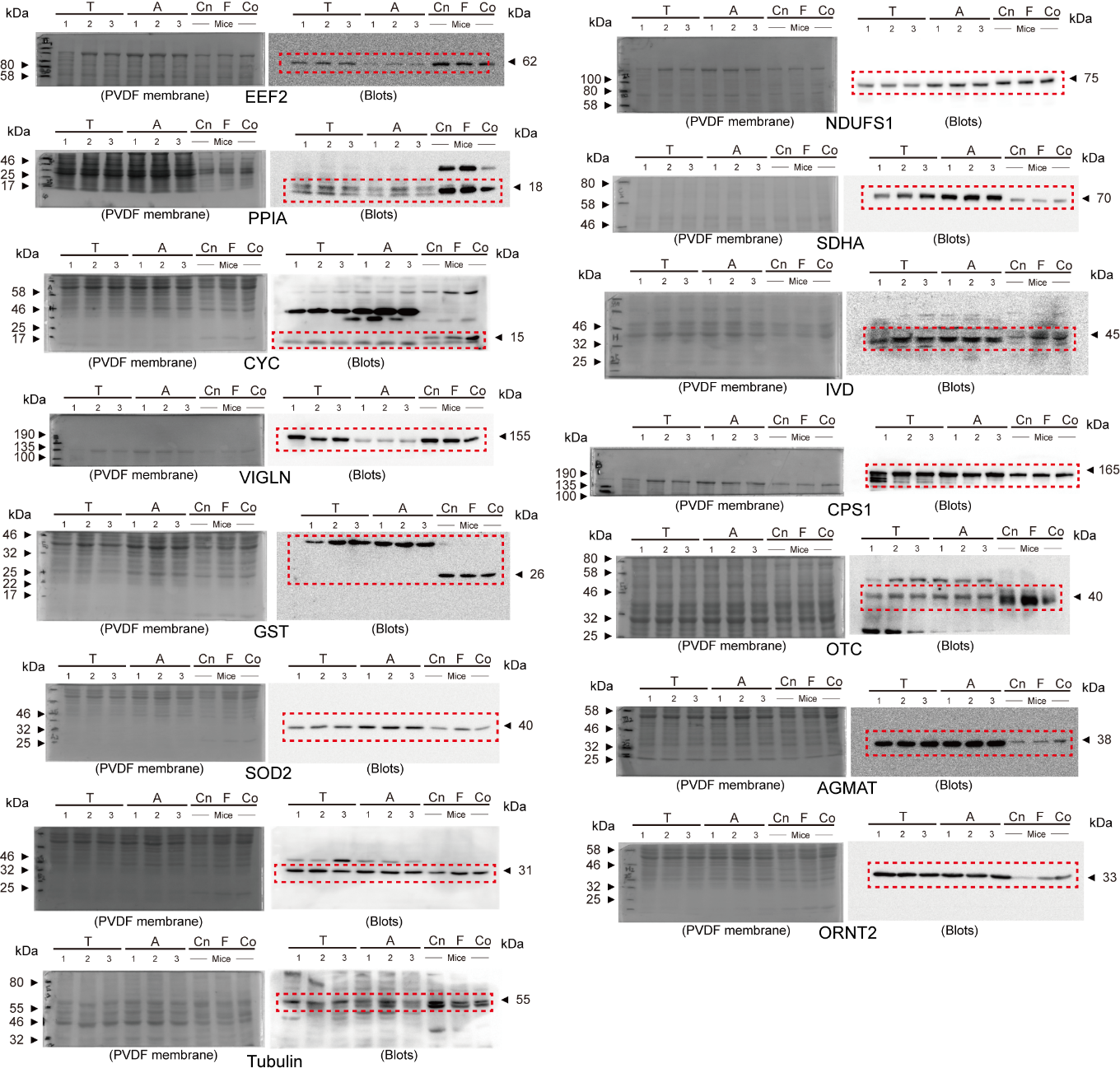


**Supplementary Fig. 7.** Ponceau-stained PVDF membranes and uncropped Western blotting images of Figure 3.

**
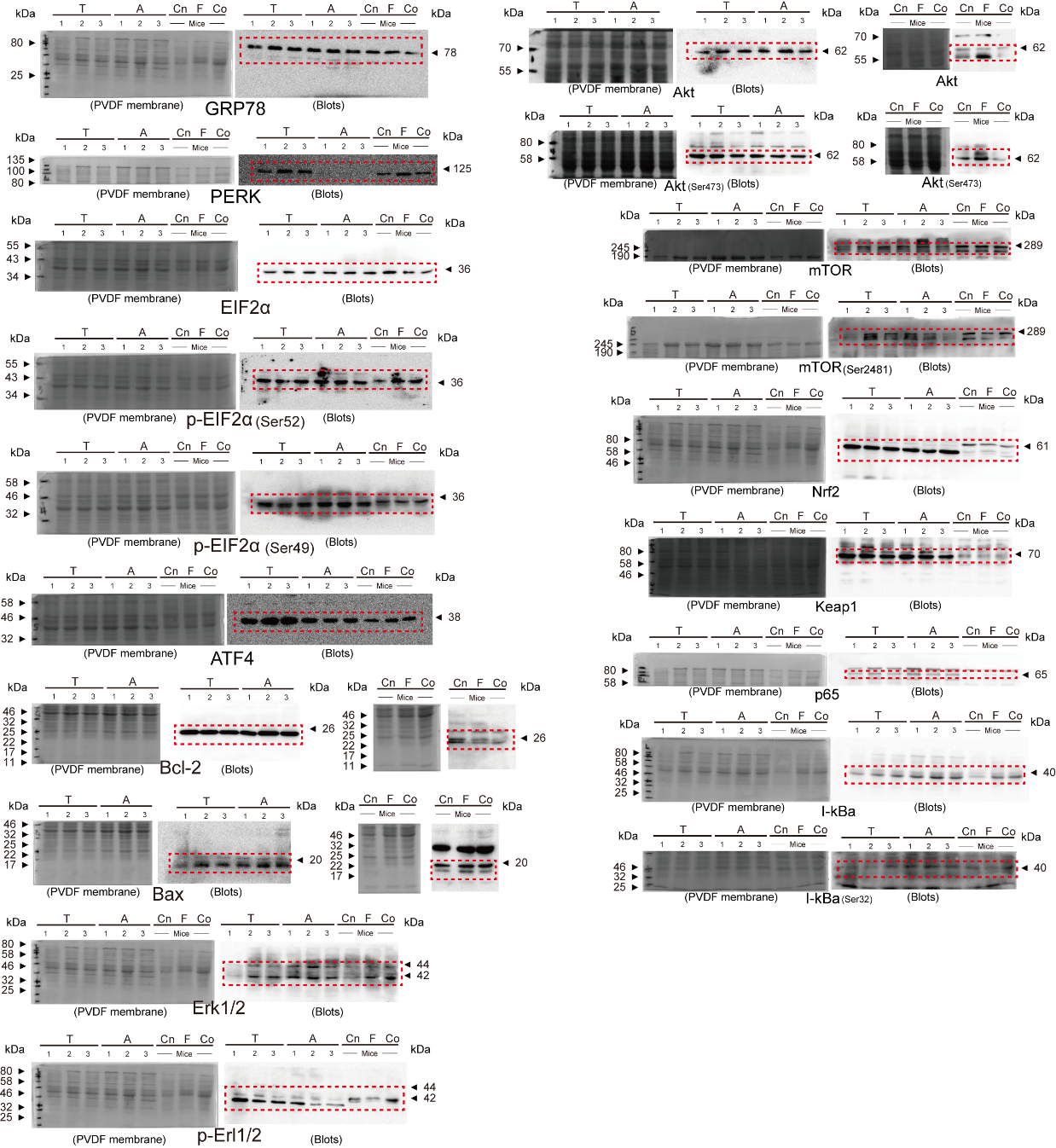
**

**Supplementary Fig. 8.** Ponceau-stained PVDF membranes and uncropped Western blotting images of Figure 4 and Figure 5.

**Supplementary Results**

***Tricarboxylic acid (TCA) cycle and electron transport chain***

Most enzymes involved in the TCA cycle had a lower abundance in bats during hibernation. However, dihydrolipoyl dehydrogenase (DLD***^b80^***), which is a component of the pyruvate dehydrogenase complex (PDHc) and α-ketoglutarate dehydrogenase complex (OGDC), and succinyl-CoA ligase [ADP-forming] subunit beta (SUCB1***^b75^***), a component of the succinyl-CoA ligase (SCS) that catalyzes the reversible conversion of succinyl-CoA to succinate, had a higher level during torpor (Fig. 3B). The levels of most subunits of mitochondrial complexes were lower during hibernation, but those of NADH dehydrogenase 1 alpha subcomplex subunit 4 (NDUFA4***^b73^***) and cytochrome c oxidase subunit 6C (COX6C***^b60^***) were increased during torpor (Fig. 3B). These results suggest that some proteins were selectively produced in response to torpor.

***Decreased glycolysis and increased gluconeogenesis in torpid bats***

Four enzymes including fructose-bisphosphate aldolase B (ALDOB***^b01^***), alpha-enolase A (ENOA***^b02^***), phosphoglycerate kinase 1 (PGK1***^b17^***), and PGK2***^b31^*** involved in glycolysis had a lower abundance in torpid and 2 h-arousal bats than in 24 h-arousal and active bats. L-lactate dehydrogenase A (LDHA***^b30^***) that catalyzes lactate and pyruvate conversions also had a lower abundance during hibernation (Fig. 3b and Supplementary Data 4). Most of the enzymes involved in gluconeogenesis, such as pyruvate carboxylase (PC***^b65^***), glucose-6-phosphate isomerase (G6PI***^b51^***), and glyceraldehyde-3-phosphate dehydrogenase 4 (GAPDH4***^b52^***), had a higher abundance in torpid than in active bats. The abundance of some enzymes involved in glycogen metabolism, such as glycogen phosphorylase (PYGL***^b53^***), glycogen debranching enzyme (GDE***^b68^***), and glycogen branching enzyme (GBE1***^b71^***), were maintained at a high level in torpid bats. These results suggest that bats have evolved the ability to use glucose during torpor (Fig. 3b and Supplementary Data 4).
