# Supplementary material for: Co-activation of Akt, Nrf2, and NF-κB signals under UPRER in torpid Myotis ricketti bats for survival
Source: Commun Biol. 2020 Nov 11;3:658. doi: 10.1038/s42003-020-01378-2 (PMC7658203; doi:10.1038/s42003-020-01378-2)
Supplement: Supplementary file 3 — Description of Additional Supplementary Files [file 42003_2020_1378_MOESM3_ESM.pdf]

Description of Additional Supplementary Files

Supplementary Data 1. Original data of volcano plot.

Supplementary Data 2. Results of IPA analysis.

Supplementary Data 3. Detailed information on heat maps in Fig. 3a.

Supplementary Data 4. Detailed information on heat maps in Fig. 3b.

Supplementary Data 5. Body weight, food intake, and rectal temperature of mice.

Supplementary Data 6. Non-overlapped protein names used in GO and IPA analysis.

Supplementary Data 7. Closeness centrality of highly connected proteins.

Supplementary Data 8. Antibodies used in this study.

Supplementary Data 9. Source data for all bar graphs.
