# Supplementary material for: Co-activation of Akt, Nrf2, and NF-κB signals under UPRER in torpid Myotis ricketti bats for survival
Source: Commun Biol. 2020 Nov 11;3:658. doi: 10.1038/s42003-020-01378-2 (PMC7658203; doi:10.1038/s42003-020-01378-2)
Supplement: Supplementary file 13 — Reporting summary [file 42003_2020_1378_MOESM13_ESM.pdf]

nature research

Corresponding author(s): Yi-Husan Pan

Last updated by author(s): Oct 3, 2020

Reporting Summary

Nature Research wishes to improve the reproducibility of the work that we publish. This form provides structure for consistency and transparency in reporting. For further information on Nature Research policies, seeAuthors & Referees and theEditorial Policy Checklist .

Statistics

For all statistical analyses, confirm that the following items are present in the figure legend, table legend, main text, or Methods section.

n/a	Confirmed	
☐	☒	The exact sample size (*n*) for each experimental group/condition, given as a discrete number and unit of measurement
☐	☒	A statement on whether measurements were taken from distinct samples or whether the same sample was measured repeatedly
☐	☒	The statistical test(s) used AND whether they are one- or two-sided<br>*Only common tests should be described solely by name; describe more complex techniques in the Methods section.*
☐	☒	A description of all covariates tested
☐	☒	A description of any assumptions or corrections, such as tests of normality and adjustment for multiple comparisons
☐	☒	A full description of the statistical parameters including central tendency (e.g. means) or other basic estimates (e.g. regression coefficient) AND variation (e.g. standard deviation) or associated estimates of uncertainty (e.g. confidence intervals)
☐	☒	For null hypothesis testing, the test statistic (e.g. *F*, *t*, *r*) with confidence intervals, effect sizes, degrees of freedom and *P* value noted<br>*Give P values as exact values whenever suitable.*
☒	☐	For Bayesian analysis, information on the choice of priors and Markov chain Monte Carlo settings
☐	☒	For hierarchical and complex designs, identification of the appropriate level for tests and full reporting of outcomes
☒	☐	Estimates of effect sizes (e.g. Cohen's *d*, Pearson's *r*), indicating how they were calculated

Our web collection on statistics for biologists contains articles on many of the points above.

Software and code

Policy information about availability of computer code

Data collection	The Xcalibur software package (version 2.0.7 SR1, Thermo-Finnigan Inc., San Jose, CA) was used to process the RAW data and the data were analyzed by PEAKS software (v. 8.5, Bioinformatics Solutions Inc., Waterloo, Ontario, Canada) and searched for the best matched peptides in our bat protein database (containing 539,834 entries) 30. All proteins identified were annotated with UniPort ID 30. The following parameters were used to analyze proteins: 20 ppm peptide mass tolerance and 0.8 Da fragment mass tolerance; precursor mass search type, monoisotopic; enzyme, trypsin; max missed cleavage, 2; nonspecific cleavage, 0; fixed modification; S-pyridylethylation; variable modification, methionine oxidation; and variable PTMs per peptide, 2. Results were adjusted to 0.5% FDR at peptide spectrum matches, -10 log P > 20, unique peptides ≥ 1, and De novo ALC score ≥ 80%. For quantitation, MS spectral counts were normalized to the sum of the spectral counts of a sample 74 (Supplementary Fig. 6). Information on identified peptides and their charge states has been submitted to ProteomeXchange (DOI: 10.6019/PXD016109 containing 160 Raw, 160 Mgf, and 1 MZID files) 75.

Data analysis	GO enrichment analysis: Non-overlapped proteins (Supplementary Data 6) were classified by Gene ontology (GO) according to their cellular component, molecular function, and biological process (Fig. 1b).<br><br>PPI analysis: The functional networks were created by NetworkAnalyst and processed with Gephi. The closeness centrality (CC) of highly connected proteins were calculated, and the top 4 hubs (CC > 0.3) in the network are presented in Fig. 2 and Supplementary Data 7.<br><br>IPA analysis: The clinical pathways of identified proteins were determined by INGENUITY® Pathway Analysis (IPA) based on significant relationships among the proteins. Statistical significance was represented by P value of overlap calculated with Fisher's exact test. The maximum false discovery rate of the pathway was < 5% when the P value threshold was < 0.05. Proteins related to survival or metabolism were selected based on GO annotation and heat maps 76 (Fig. 3, Supplementary Data 3 and 4).<br><br>Other analysis: Several R packages (venn, FactoMineR and pheatmap), were used for Venn Plots, PCA analysis, and heat map display.

For manuscripts utilizing custom algorithms or software that are central to the research but not yet described in published literature, software must be made available to editors/reviewers. We strongly encourage code deposition in a community repository (e.g. GitHub). See the Nature Research guidelines for submitting code & software for further information.

Data

Policy information about availability of data

All manuscripts must include a data availability statement. This statement should provide the following information, where applicable:

- Accession codes, unique identifiers, or web links for publicly available datasets
- A list of figures that have associated raw data
- A description of any restrictions on data availability

The mass spectrometry proteomics data of this study have been deposited to the ProteomeXchange; Project Name: Co-activation of Akt, Nrf2, and NF-κB signaling pathways via the PERK-EIF2-ATF4 regulatory axis under UPRER as a mechanism of survival for torpid Myotis ricketti bats; Project accession: PXD016109; Project DOI: 10.6019/PXD016109; Reviewer account details: Username: reviewer02588@ebi.ac.uk; Password: JA1atSfn.

Field-specific reporting

Please select the one below that is the best fit for your research. If you are not sure, read the appropriate sections before making your selection.

☒ Life sciences ☐ Behavioural & social sciences ☐ Ecological, evolutionary & environmental sciences

For a reference copy of the document with all sections, see nature.com/documents/nr-reporting-summary-flat.pdf

Life sciences study design

All studies must disclose on these points even when the disclosure is negative.

Sample size	Sixteen bats were used in this study and twelve 11-week-old mice were obtained from Sino-British Sippr/BK Lab Animal Ltd (Shanghai, China), maintained in a 12-h dark-light cycle at 28°C , and provided food and water ad libitum.
Data exclusions	No data were excluded from the analyses.
Replication	For each experiment, at least three technical repeats for each animal individual were performed and all values were used in calculation.
Randomization	Mice were randomly divided into 3 groups (4 mice/group): (1) ad libitum to food at 27°C (control group), (2) ad libitum to food at 20°C (cold-stimulated group), and (3) fasting at 27°C (fasted group).
Blinding	Because all bats and mice used were separately divided by their physiological states or treatments, so that each tissue sample was carefully obtained and preserved with clear tag on the plastic vial. Therefore, it is not possible for us to ignore the labeling. However, we guarantee our faithful calculation and presentation in this work.

Reporting for specific materials, systems and methods

We require information from authors about some types of materials, experimental systems and methods used in many studies. Here, indicate whether each material, system or method listed is relevant to your study. If you are not sure if a list item applies to your research, read the appropriate section before selecting a response.

Materials & experimental systems

n/a	Involved in the study	
☐	☒	Antibodies
☒	☐	Eukaryotic cell lines
☒	☐	Palaeontology
☐	☒	Animals and other organisms
☒	☐	Human research participants
☒	☐	Clinical data

Methods

n/a	Involved in the study	
☒	☐	ChIP-seq
☒	☐	Flow cytometry
☒	☐	MRI-based neuroimaging

Antibodies

Antibodies used

All antibodies used in the study were described in Supplementary Data 8. The catalog number, supplier name, loading quantity, and conditions used for each primary or secondary antibody were all clearly provided.

Validation

All Conditions for the use of antibody were listed in Supplementary Data 8 and these antibodies were chosen because they recognized conserved epitopes across many mammalian species. The quality of each of them was also tested in mice and bats before applied in the formal experiments.

Animals and other organisms

Policy information about studies involving animals; ARRIVE guidelines recommended for reporting animal research

Laboratory animals

Twelve 11-week-old ICR male mice were obtained from Sino-British Sippr/BK Lab Animal Ltd (Shanghai, China), maintained in a 12-h dark-light cycle at 28oC, and provided food and water ad libitum. Mice of this age were chosen as their torpor-like states had been successfully induced 69-71. Mice were randomly divided into 3 groups (4 mice/group) at the seventh day after arrival: (1) ad libitum to food at 27oC (control group), (2) ad libitum to food at 20oC (cold-stimulated group), and (3) fasting at 27oC (fasted group). Each condition was maintained for 2 days with water freely available 73. Body weight, food intake, and body temperature of all mice were recorded at the first day and every two days (Supplementary Fig. 4 and Supplementary Data 5). All animals used in this study were sacrificed by cervical dislocation, and their livers were rapidly removed, snap frozen in liquid nitrogen, and stored at -80°C until used.

Wild animals

Myotis ricketti bats were captured from Fangshan cave (39°48'N, 115°42'E), Beijing, China. The temperature inside the caves was approximately 10°C, and the ambient temperature outside the cave was below 4°C. Because female bats are often pregnant during hibernation season, only adult male bats were used. Twelve male torpid bats were captured using hand nets. Four of them were sacrificed immediately. Each of the remaining 8 bats was placed separately in a cloth bag and transported within 50 min to the lab, where the temperature was maintained at 28°C. These bats were spontaneously aroused during transportation. Four bats were sacrificed 2 hours after arousal, and the other 4 bats were sacrificed 24 hours after arousal. Four active male bats were also captured in summer from the same cave; these bats were sacrificed immediately upon capture. The average surface and rectal temperatures were 8°C and 11°C for torpid bats, 29°C and 31°C for 2-hr aroused bats, 32°C and 35°C for 24-hr aroused bats, and 35oC and 36oC for active bats, respectively.

Field-collected samples

Myotis ricketti bats were captured from Fangshan cave (39°48'N, 115°42'E), Beijing, China. The temperature inside the caves was approximately 10°C, and the ambient temperature outside the cave was below 4°C.

Ethics oversight

Animals used in this study are not endangered or protected species. All experiments were approved by the Animal Ethics Committee of East China Normal University (approval number AR2012/03001).

Note that full information on the approval of the study protocol must also be provided in the manuscript.
